# Density by moduli and Wijsman lacunary statistical convergence of sequences of sets

**DOI:** 10.1186/s13660-017-1294-2

**Published:** 2017-01-23

**Authors:** Vinod K Bhardwaj, Shweta Dhawan

**Affiliations:** 1 Department of Mathematics, Kurukshetra University, Kurukshetra, 136119 India; 2 Department of Mathematics, KVA DAV College for Women, Karnal, 132001 India

**Keywords:** 40A35, 46A45, 40G15, modulus function, density, lacunary sequence, statistical convergence, lacunary strong convergence, Wijsman convergence

## Abstract

The main object of this paper is to introduce and study a new concept of *f*-Wijsman lacunary statistical convergence of sequences of sets, where *f* is an unbounded modulus. The definition of Wijsman lacunary strong convergence of sequences of sets is extended to a definition of Wijsman lacunary strong convergence with respect to a modulus for sequences of sets and it is shown that, under certain conditions on a modulus *f*, the concepts of Wijsman lacunary strong convergence with respect to a modulus *f* and *f*-Wijsman lacunary statistical convergence are equivalent on bounded sequences. We further characterize those *θ* for which $\mathit{WS}_{\theta}^{f} = \mathit{WS}^{f}$, where $\mathit{WS}_{\theta}^{f}$ and $\mathit{WS}^{f}$ denote the sets of all *f*-Wijsman lacunary statistically convergent sequences and *f*-Wijsman statistically convergent sequences, respectively.

## Introduction

Zygmund [1] was the person behind the introduction of the idea of statistical convergence. The concept of statistical convergence was formally given by Fast [2] and Steinhaus [3]. This concept was studied by Schoenberg [4] as a non-matrix summability method. For a detailed account of statistical convergence one may refer to [5–11], and many others.

Let $\mathbb{N}$ denote the set of all natural numbers. A number sequence $X=(\xi_{k})$ is said to be statistically convergent to the number *l* if for each $\varepsilon>0$ the set $\{k \in\mathbb{N}: \vert \xi_{k} - l\vert \geq\varepsilon\}$ has natural density zero. The natural density of a subset $E \subset\mathbb{N}$ [12] is defined by

$$d(E) = \lim_{n \to\infty}\frac{1}{n} \bigl\vert \{ k\leq n : k \in E \} \bigr\vert , $$

where the vertical bars indicate the number of elements in the enclosed set. Obviously we have $d(E) =0$ provided that *E* is a finite set of positive integers. If a sequence $(\xi_{k})$ is statistically convergent to *l*, then we write it as $S-\lim\xi_{k} = l$ or $\xi_{k}\to l(S)$.

The concept of convergence of sequences of points has been extended by several authors [13–20] to the convergence of sequences of sets. In this paper we consider one such extension, namely, Wijsman convergence. Nuray and Rhoades [21] extended the notion of Wijsman convergence of sequences of sets to that of Wijsman statistical convergence of sequences of sets, and gave some basic theorems. Also, they introduced the notion of Wijsman strong Cesàro summability of sequences of sets and discussed its relation with Wijsman statistical convergence. Ulusu and Nuray [22] introduced the concepts of Wijsman lacunary statistical convergence of sequences of sets and Wijsman lacunary strong convergence of sequences of sets and established a relation between them. For more work on convergence of sequences of sets one may refer to [21–30].

Recall [31, 32] that a modulus *f* is a function from $\mathbb{R^{+}}$ to $\mathbb{R^{+}}$ such that

(i) $f(x) = 0$ if and only if $x = 0$,
(ii) $f(x + y) \leq f(x) + f(y)$ for $x \geq0$, $y \geq 0$,
(iii) *f* is increasing,
(iv) *f* is continuous from the right at 0.

From above properties it is easy to see that a modulus *f* is continuous on $\mathbb{R^{+}}$. A modulus may be unbounded or bounded. For example, $f(x) = x^{p}$ where $0 < p \leq1$, is unbounded, but $f(x) = \frac{x}{ (1+ x)}$ is bounded. The work related to the sequence spaces defined by a modulus may be found in, *e.g.*, [24, 31, 33–37].

Aizpuru *et al.* [33] have recently introduced a new concept of density by moduli and consequently obtained a new concept of non-matrix convergence, namely, *f*-statistical convergence which is, in fact, a generalization of the concept of statistical convergence and intermediate between the ordinary convergence and the statistical convergence. This idea of replacing natural density with density by moduli has motivated us to look for some new generalizations of statistical convergence and consequently we have introduced and studied the concepts of *f*-statistical convergence of order *α* [34] and *f*-lacunary statistical convergence [35]. Using the notion of density by moduli Bhardwaj *et al.* [36] have also introduced and studied the concept of *f*-statistical boundedness which is a generalization of statistical boundedness [38] and intermediate between the usual boundedness and the statistical boundedness.

The notion of Wijsman statistical convergence has been extended by Bhardwaj *et al.* to that of *f*-Wijsman statistical convergence [unpublished], where *f* is an unbounded modulus.

Before proceeding further, we first recall some definitions.

### Definition 1.1

[33]

For any unbounded modulus *f*, the *f*-density of a set $E \subset \mathbb{N}$ is denoted by $d^{f} (E)$ and is defined by

$$d^{f} (E) = \lim_{n \to\infty} \frac{f (\vert \{k \leq n: k \in E \}\vert )}{f(n)} $$

in the case this limit exists. Clearly, finite sets have zero *f*-density and $d^{f} (\mathbb{N} -E) = 1- d^{f} (E) $ does not hold, in general. But if $d^{f}(E)=0$ then $d^{f}(\mathbb{N}-E) = 1$.

### Remark 1.2

For any unbounded modulus *f*, if $E \subset\mathbb{N}$ has zero *f*-density then it has zero natural density, however, the converse need not be true. For example, if we take $f(x)= \log{(x+1)}$ and $E = \{ n^{2} : n \in\mathbb{N} \}$, then $d(E) = 0$ but $d^{f}(E) = 1/2 $.

### Definition 1.3

[33]

Let *f* be an unbounded modulus. A number sequence $X=(\xi_{k})$ is said to be *f*-statistically convergent to *l*, or $S^{f}$-convergent to *l*, if, for each $\varepsilon > 0$,

$$\begin{aligned} &d^{f} \bigl( \bigl\{ k \in\mathbb{N}: \vert \xi_{k} - l \vert \geq \varepsilon \bigr\} \bigr) = 0, \\ &\text{i.e.,} \quad \lim_{n \to\infty}\frac{1}{f(n)}f \bigl( \bigl\vert \bigl\{ k \leq n : \vert \xi_{k} - l\vert \geq \varepsilon \bigr\} \bigr\vert \bigr) =0, \end{aligned}$$

and we write it as $S^{f}-\lim \xi_{k} = l$ or $\xi_{k} \to l(S^{f})$.

It is an immediate consequence of Definition 1.3 and Remark 1.2 that every *f*-statistically convergent sequence is statistically convergent, but a statistically convergent sequence need not be *f*-statistically convergent for every unbounded modulus *f*.

By a lacunary sequence $\theta= (k_{r})$; $r = 0,1,2,\ldots$ , where $k_{o} = 0$, we shall mean an increasing sequence of non-negative integers with $h_{r} = k_{r} - k_{r-1} \to \infty$ as $r \to\infty$. The intervals determined by *θ* will be denoted by $I_{r} = ( k_{r-1}, k_{r}]$ and the ratio $k_{r}/k_{r-1}$ will be denoted by $q_{r}$.

The space of all lacunary strongly convergent sequences, $N_{\theta}$, was defined by Freedman *et al.* [39] as follows:

$$ N_{\theta} = \biggl\{ X = (\xi_{k}) : \lim_{r \to \infty} \frac{1}{h_{r}}\sum_{k \in I_{r}}\vert \xi_{k} - l \vert = 0 \text{ for some number } l \biggr\} . $$

There is a strong connection [39] between $N_{\theta}$ and the space *w* of strongly Cesàro summable sequences, which is defined by

$$ w = \Biggl\{ X = (\xi_{k}) : \lim_{n \to \infty} \frac{1}{n}\sum_{k = 1}^{n}\vert \xi_{k} - l \vert = 0 \text{ for some number } l \Biggr\} . $$

In the special case, where $\theta= (2^{r})$, we have $N_{\theta}= w$.

In the year 1986, the concept of strong Cesàro summability was extended to that of strong Cesàro summability with respect to a modulus by Maddox [31]. A sequence $X = (\xi_{k})$ is said to be strongly Cesàro summable with respect to a modulus *f* to *l* if

$$\lim_{n \to\infty}\frac{1}{n}\sum_{k = 1}^{n}f \bigl(\vert \xi_{k} - l \vert \bigr) = 0. $$

The space of strongly Cesàro summable sequences with respect to a modulus *f*, is denoted by $w(f)$.

Furthermore, in the year 1994, Pehlivan and Fisher [40] extended the notion of lacunary strong convergence to that of lacunary strong convergence with respect to a modulus *f*. The space $N_{\theta}(f)$ of lacunary strongly convergent sequences with respect to a modulus *f* is defined as

$$ N_{\theta}(f) = \biggl\{ X = (\xi_{k}) : \lim _{r \to \infty}\frac{1}{h_{r}}\sum_{k \in I_{r}}f \bigl(\vert \xi_{k} - l \vert \bigr) = 0 \text{ for some number } l \biggr\} . $$

Fridy and Orhan [9] introduced the concept of lacunary statistical convergence as follows.

### Definition 1.4

Let $\theta= (k_{r})$ be a lacunary sequence. A number sequence $X=(\xi_{k})$ is said to be lacunary statistically convergent to *l*, or $S_{\theta}$-convergent to *l*, if, for each $\varepsilon > 0$,

$$\lim_{r \to\infty}\frac{1}{h_{r}} \bigl\vert \bigl\{ k \in I_{r} : \vert \xi_{k} - l\vert \geq\varepsilon \bigr\} \bigr\vert =0. $$

In this case, we write $S_{\theta}-\lim \xi_{k} = l$ or $\xi_{k} \to l(S_{\theta})$.

Quite recently, Ulusu and Nuray [22] introduced the notion of Wijsman lacunary strong convergence of sequences of sets and discussed its relation with Wijsman lacunary statistical convergence.

In this paper, we first extend the definition of Wijsman lacunary strong convergence to a definition of Wijsman lacunary strong convergence with respect to a modulus. It is shown that if a sequence is Wijsman lacunary strongly convergent then it is Wijsman lacunary strongly convergent with respect to a modulus, however, the converse need not be true. We also investigate the condition under which the converse is true. We also study a relationship between Wijsman lacunary strong convergence with respect to a modulus and Wijsman lacunary statistical convergence and characterize those *θ* for which $[\mathit {Ww}^{f}] = [\mathit {WN}_{\theta}^{f}]$, where $[\mathit {Ww}^{f}]$ is the set of all Wijsman strongly Cesàro summable sequences with respect to a modulus *f* and $[\mathit {WN}_{\theta}^{f}]$ is the set of all Wijsman lacunary strongly convergent sequences with respect to a modulus *f*. We also introduce a new concept of *f*-Wijsman lacunary statistical convergence of sequences of sets which is a generalization of the concept of Wijsman lacunary statistical convergence of sequences of sets and intermediate between the usual Wijsman convergence and the Wijsman lacunary statistical convergence. It is proved that, under certain conditions on the modulus *f*, the concepts of Wijsman lacunary strong convergence with respect to a modulus *f* and *f*-Wijsman lacunary statistical convergence are equivalent on bounded sequences of sets. We also characterize those *θ* for which $\mathit{WS}^{f} = \mathit{WS}_{\theta}^{f}$, under certain restrictions on unbounded modulus *f*, where $\mathit{WS}^{f}$ is the set of all *f*-Wijsman statistically convergent sequences of sets and $\mathit{WS}_{\theta}^{f}$ is the set of all *f*-Wijsman lacunary statistically convergent sequences of sets. Finally, we observe that it is possible for a sequence to have different $\mathit{WS}_{\theta}^{f}$-limits for different *θ*’s. In Theorem 4.1, we investigate certain conditions under which this situation cannot occur.

Before proceeding to establish the proposed results, we pause to collect some definitions related to Wijsman convergence [21, 22].

Let $(M,\rho)$ be a metric space. The distance $d(x,E)$ from a point *x* to a non-empty subset *E* of $(M,\rho)$ is defined to be

$$d(x,E)= \inf_{y \in E}\rho(x,y). $$

### Definition 1.5

Let $(E_{k})$ be a sequence of non-empty closed subsets of a metric space $(M,\rho)$ and *E* be non-empty closed subset of *M*.

- $(E_{k})$ is said to be Wijsman convergent to *E*, if, for each $x \in M$, $(d(x,E_{k}))$ is convergent to $d(x,E)$. In this case, we write $Wc-\lim E_{k} = E$ or $E_{k} \to E(Wc)$. The set of all Wijsman convergent sequences is denoted by *Wc*.
- $(E_{k})$ is said to be Wijsman statistically convergent to *E*, or *WS*-convergent to *E*, if, for each $x \in M$, $(d(x,E_{k}))$ is statistically convergent to $d(x,E)$; *i.e.*, for each $x \in M$ and for each $\varepsilon > 0$,
  $$\lim_{n \to\infty}\frac{1}{n} \bigl\vert \bigl\{ k \leq n : \bigl\vert d(x,E_{k}) - d(x,E) \bigr\vert \geq \varepsilon \bigr\} \bigr\vert =0. $$
  In this case, we write $\mathit{WS}-\lim E_{k} = E$ or $E_{k} \to E(\mathit{WS})$. The set of all sequences which are Wijsman statistically convergent is denoted by *WS*.
- $(E_{k})$ is said to be bounded if $\sup_{k} \vert d(x,E_{k})\vert < \infty$ for each $x \in M$. We shall denote the set of all bounded sequences of sets by $L_{\infty}^{\prime}$.
- $(E_{k})$ is said to be Wijsman strongly Cesàro summable to *E*, if, for each $x \in M$, $(d(x,E_{k}))$ is strongly Cesàro summable to $d(x,E)$; *i.e.*, for each $x \in M$,
  $$\lim_{n \to\infty}\frac{1}{n}\sum_{k=1}^{n} \bigl\vert d(x,E_{k})-d(x,E) \bigr\vert =0. $$
  In this case, we write $[\mathit {Ww}]-\lim E_{k} = E$ or $E_{k} \to E[\mathit {Ww}]$. The set of all sequences which are Wijsman strongly Cesàro summable is denoted by $[\mathit {Ww}]$.
- $(E_{k})$ is said to be Wijsman lacunary strongly convergent to *E*, if, for each $x \in M$, $(d(x,E_{k}))$ is lacunary strongly convergent to $d(x,E)$; *i.e.*, for each $x \in M$,
  $$\lim_{r \to\infty}\frac{1}{h_{r}}\sum_{k \in I_{r}} \bigl\vert d(x,E_{k})-d(x,E) \bigr\vert =0. $$
  In this case, we write $[\mathit {WN}_{\theta}]-\lim E_{k} = E$ or $E_{k} \to E[\mathit {WN}_{\theta}]$. The set of all sequences which are Wijsman lacunary strongly convergent is denoted by $[\mathit {WN}_{\theta}]$.
- $(E_{k})$ is said to be Wijsman lacunary statistically convergent to *E*, or $\mathit{WS}_{\theta}$-convergent to *E*, if, for each $x \in M$, $(d(x,E_{k}))$ is lacunary statistically convergent to $d(x,E)$; *i.e.*, for each $x \in M$ and for each $\varepsilon > 0$,
  $$\lim_{r \to\infty}\frac{1}{h_{r}} \bigl\vert \bigl\{ k \in I_{r} : \bigl\vert d(x,E_{k}) - d(x,E) \bigr\vert \geq \varepsilon \bigr\} \bigr\vert =0. $$
  In this case, we write $\mathit{WS}_{\theta}-\lim E_{k} = E$ or $E_{k} \to E(\mathit{WS}_{\theta})$. The set of all sequences which are Wijsman lacunary statistically convergent is denoted by $\mathit{WS}_{\theta}$.

Quite recently, Bhardwaj *et al.* have given the following definitions [unpublished].

### Definition 1.6

For any an unbounded modulus *f*, a sequence $(E_{k})$ of non-empty closed subsets of a metric space $(M,\rho)$ is said to be *f*-Wijsman statistically convergent to a non-empty closed subset *E* of M, or $\mathit{WS}^{f}$-convergent to *E*, if, for each $x \in M$, $(d(x,E_{k}))$ is *f*-statistically convergent to $d(x,E)$; *i.e.*, for each $x \in M$ and for each $\varepsilon > 0$,

$$\lim_{n \to\infty}\frac{1}{f(n)}f \bigl( \bigl\vert \bigl\{ k \leq n : \bigl\vert d(x,E_{k}) - d(x,E) \bigr\vert \geq \varepsilon \bigr\} \bigr\vert \bigr) =0. $$

In this case, we write $\mathit{WS}^{f}-\lim E_{k} = E$ or $E_{k} \to E(\mathit{WS}^{f})$. The set of all sequences which are *f*-Wijsman statistically convergent is denoted by $\mathit{WS}^{f}$.

### Definition 1.7

For any modulus *f*, a sequence $(E_{k})$ of non-empty closed subsets of a metric space $(M,\rho)$ is said to be Wijsman strongly Cesàro summable to a non-empty closed subset *E* of *M* with respect to *f*, if, for each $x \in M$, $(d(x,E_{k}))$ is strongly Cesàro summable to $d(x,E)$ with respect to *f*; *i.e.*, for each $x \in M$,

$$\lim_{n \to\infty}\frac{1}{n}\sum_{k=1}^{n}f \bigl( \bigl\vert d(x,E_{k}) - d(x,E) \bigr\vert \bigr) =0. $$

In this case, we write $[\mathit {Ww}^{f}]-\lim E_{k} = E$ or $E_{k} \to E[\mathit {Ww}^{f}]$. The set of all sequences which are Wijsman strongly Cesàro summable with respect to a modulus *f* is denoted by $[\mathit {Ww}^{f}]$.

## Wijsman lacunary strong convergence with respect to a modulus

### Definition 2.1

Let $(M,\rho)$ be a metric space, *f* be a modulus and $\theta= (k_{r})$ be a lacunary sequence. A sequence $(E_{k})$ of non-empty closed subsets of M is said to be Wijsman lacunary strongly convergent to a non-empty closed subset *E* of *M* with respect to *f*, if, for each $x \in M$, $(d(x,E_{k}))$ is lacunary strongly convergent to $d(x,E)$ with respect to *f*; *i.e.*, for each $x \in M$,

$$\lim_{r \to\infty}\frac{1}{h_{r}}\sum_{k \in I_{r}}f \bigl( \bigl\vert d(x,E_{k}) - d(x,E) \bigr\vert \bigr) =0. $$

In this case, we write $[\mathit {WN}_{\theta}^{f}]-\lim E_{k} = E$ or $E_{k} \to E[\mathit {WN}_{\theta}^{f}]$. The set of all sequences which are Wijsman lacunary strongly convergent with respect to a modulus *f* is denoted by $[\mathit {WN}_{\theta}^{f}]$.

### Remark 2.2

If we take $f(x) = x$, the concept of Wijsman lacunary strong convergence with respect to *f* reduces to that of Wijsman lacunary strong convergence.

We now study an inclusion relation between $[\mathit {WN}_{\theta}]$ and $[\mathit {WN}_{\theta}^{f}]$.

To establish this relation we first recall the following proposition from [40].

### Proposition 2.3

*Let* *f* *be a modulus and let* $0 < \delta<1$. *Then*, *for each* $x \geq \delta$, *we have* $f(x) \leq2f(1)\delta^{-1}x$.

### Theorem 2.4

*For any modulus* *f*, *we have* $[\mathit {WN}_{\theta}] \subset[\mathit {WN}_{\theta}^{f}]$.

### Proof

Let $(E_{k}) \in[\mathit {WN}_{\theta}] $, then, for each $x\in M$,

$$N_{r} = \frac{1}{h_{r}}\sum_{k \in I_{r}} \bigl\vert d(x,E_{k}) - d(x,E) \bigr\vert \to0 \quad (\text{as } r \to\infty). $$

Let $\varepsilon> 0$ be given. We choose $0 < \delta< 1$ such that $f(u) < \varepsilon$ for every *u* with $0 \leq u \leq\delta$. We can write

$$\begin{aligned} &\frac{1}{h_{r}}\sum_{k \in I_{r}} f \bigl( \bigl\vert d(x,E_{k}) - d(x,E) \bigr\vert \bigr) \\ &\quad =\frac{1}{h_{r}} \biggl( \mathop{\sum_{k \in I_{r}}}_{\vert d(x,E_{k}) - d(x,E)\vert \leq\delta}f \bigl( \bigl\vert d(x,E_{k}) - d(x,E) \bigr\vert \bigr) \biggr) \\ &\quad\quad{} +\frac{1}{h_{r}} \biggl( \mathop{\sum_{k \in I_{r}}}_{\vert d(x,E_{k}) - d(x,E)\vert > \delta}f \bigl( \bigl\vert d(x,E_{k}) - d(x,E) \bigr\vert \bigr) \biggr) \\ &\quad \leq\frac{1}{h_{r}}(h_{r}\varepsilon) + 2f(1)\delta^{-1}N_{r}, \end{aligned}$$

by Proposition 2.3. Therefore, $(E_{k}) \in[\mathit {WN}_{\theta}^{f}]$ as $r \to\infty$.

### Remark 2.5

The converse of the above theorem does not need to be true, which can be verified from the following example.

### Example 2.6

Let $M=\mathbb{R}$, $\rho(x,y) = \vert x-y\vert $ and $f(x) = \log(x+1) $. Consider the sequence $(E_{k})$ defined by

$$E_{k}= \textstyle\begin{cases} \{h_{r}\} , &\text{if $k \in I_{r}$ such that $k = k_{r-1}+1$,} \\ \{0\}, &\text{otherwise}. \end{cases} $$

Note that $(E_{k})$ is not a bounded sequence. Then, for each $x \in M$,

$$\begin{aligned} \frac{1}{h_{r}}\sum_{k \in I_{r}} f \bigl( \bigl\vert d(x,E_{k}) - d \bigl(x,\{0\} \bigr) \bigr\vert \bigr)&= \frac{1}{h_{r}} f \bigl( \bigl\vert d(x,E_{k_{r-1}+1}) - d \bigl(x,\{0\} \bigr) \bigr\vert \bigr) \\ &= \frac{1}{h_{r}} f \bigl( \bigl\vert d \bigl(x,\{h_{r}\} \bigr) - d \bigl(x,\{0\} \bigr) \bigr\vert \bigr) \\ &\leq\frac{1}{h_{r}} f \bigl( \bigl\vert (x-h_{r}) - (x-0) \bigr\vert \bigr) \\ &=\frac{f(h_{r})}{h_{r}} = \frac{\log(h_{r} +1)}{h_{r}} \to0 \quad (\text{as } r \to \infty), \end{aligned}$$

and so $(E_{k}) \in[\mathit {WN}_{\theta}^{f}]$, but, for $x =0$,

$$\begin{aligned} \frac{1}{h_{r}}\sum_{k \in I_{r}} \bigl\vert d(x,E_{k}) - d \bigl(x,\{0\} \bigr) \bigr\vert &= \frac{1}{h_{r}} \bigl\vert d(x,E_{k_{r-1}+1}) - d \bigl(x,\{0\} \bigr) \bigr\vert \\ &= \frac{1}{h_{r}} \bigl\vert \vert x-h_{r}\vert -\vert x-0 \vert \bigr\vert \\ &=\frac{1}{h_{r}}h_{r} \to1\quad (\text{as } r \to\infty), \end{aligned}$$

and so $(E_{k}) \notin[\mathit {WN}_{\theta}]$. □

Maddox [41] proved that for any modulus *f* there exists $\lim_{t \to\infty}\frac{f(t)}{t}$. Making use of this result we are in a position to give a condition on modulus *f* under which the converse holds.

### Theorem 2.7

*Let* *f* *be a modulus such that* $\lim_{t \to\infty}\frac{f(t)}{t} > 0$, *then* $[\mathit {WN}_{\theta}^{f}] \subset[\mathit {WN}_{\theta}]$.

The proof can be established using the technique of Theorem 3.5 of [34].

We now establish a relationship between Wijsman lacunary strong convergence with respect to a modulus and Wijsman lacunary statistical convergence.

### Theorem 2.8

*For any modulus* *f*, *we have* $[\mathit {WN}_{\theta}^{f}] \subset \mathit{WS}_{\theta}$.

### Proof

Suppose that $(E_{k}) \in[\mathit {WN}_{\theta}^{f}]$. For each $x \in M$ and $\varepsilon> 0$, we have

$$\begin{aligned} \frac{1}{h_{r}}\sum_{k \in I_{r}} f \bigl( \bigl\vert d(x,E_{k}) - d(x,E) \bigr\vert \bigr) &\geq\frac{1}{h_{r}} \biggl( \mathop{\sum_{k \in I_{r}}}_{\vert d(x,E_{k}) - d(x,E)\vert \geq\varepsilon}f \bigl( \bigl\vert d(x,E_{k}) - d(x,E) \bigr\vert \bigr) \biggr) \\ &\geq\frac{1}{h_{r}} \bigl\vert \bigl\{ k \in I_{r} : \bigl\vert d(x,E_{k}) - d(x,E) \bigr\vert \geq\varepsilon \bigr\} \bigr\vert f( \varepsilon), \end{aligned}$$

from which it follows that $(E_{k}) \in \mathit{WS}_{\theta}$. □

We now give an example to show that the converse of the above inclusion need not hold.

### Example 2.9

Let $M= \mathbb{R}$, $\rho(x,y) = \vert x-y\vert $ and $f(x) = 2x$. Consider the sequence $(E_{k})$ of subsets of *M* as defined in Example 2.6.

This sequence is Wijsman lacunary statistically convergent to the set $E = \{0\}$ because for each $x \in M$ and for each $\varepsilon> 0$,

$$\lim_{r \to\infty}\frac{1}{h_{r}} \bigl\vert \bigl\{ k \in I_{r} : \bigl\vert d(x,E_{k}) - d \bigl(x,\{0\} \bigr) \bigr\vert \geq \varepsilon \bigr\} \bigr\vert = \lim_{r \to\infty} \frac{1}{h_{r}} =0. $$

But this is not Wijsman lacunary strongly convergent with respect to *f*.

In the next theorem, we investigate a necessary and sufficient condition on *f* under which the converse holds.

### Theorem 2.10

$\mathit{WS}_{\theta} = [\mathit {WN}_{\theta}^{f}]$ *if and only if* *f* *is bounded*.

### Proof

Suppose that *f* is bounded and $(E_{k}) \in \mathit{WS}_{\theta}$. Since *f* is bounded, there exists a constant *H* such that $f(x) \leq H$ for all $x \geq0$. Now for each $x \in M$,

$$\begin{aligned} &\frac{1}{h_{r}}\sum_{k \in I_{r}} f \bigl( \bigl\vert d(x,E_{k}) - d(x,E) \bigr\vert \bigr) \\ &\quad =\frac{1}{h_{r}} \biggl( \mathop{\sum_{k \in I_{r}}}_{\vert d(x,E_{k}) - d(x,E)\vert \geq\varepsilon}f \bigl( \bigl\vert d(x,E_{k}) - d(x,E) \bigr\vert \bigr) \biggr) \\ &\quad\quad{} +\frac{1}{h_{r}} \biggl( \mathop{\sum_{k \in I_{r}}}_{\vert d(x,E_{k}) - d(x,E)\vert < \varepsilon}f \bigl( \bigl\vert d(x,E_{k}) - d(x,E) \bigr\vert \bigr) \biggr) \\ &\quad \leq\frac{1}{h_{r}} \bigl\vert \bigl\{ k \in I_{r} : \bigl\vert d(x,E_{k}) - d(x,E) \bigr\vert \geq\varepsilon \bigr\} \bigr\vert H+ \frac {1}{h_{r}}h_{r}f(\varepsilon). \end{aligned}$$

Taking the limit as $r \to\infty$, we have $(E_{k}) \in[\mathit {WN}_{\theta }^{f}]$.

Conversely, suppose that *f* is unbounded so that there exists a positive sequence $0 < p_{1} < p_{2} <\cdots< p_{i} < \cdots$ such that $f(p_{i}) \geq h_{i}$. Define the sequence $(E_{k})$ such that $E_{k_{i}} = \{ p_{i}\}$ for $i = 1,2, \ldots$ and $E_{k} = \{0\}$ otherwise. Then, for each $x \in M$ and $\varepsilon> 0$,

$$\frac{1}{h_{r}} \bigl\vert \bigl\{ k \in I_{r} : \bigl\vert d(x,E_{k}) - d \bigl(x,\{0\} \bigr) \bigr\vert \geq\varepsilon \bigr\} \bigr\vert = \frac{1}{h_{r}} \to0 \quad (\text{as } r \to\infty), $$

and so $(E_{k}) \in \mathit{WS}_{\theta}$, but, for $x = 0$,

$$\begin{aligned} \frac{1}{h_{r}}\sum_{k \in I_{r}} f \bigl( \bigl\vert d(x,E_{k}) - d \bigl(x,\{0\} \bigr) \bigr\vert \bigr) &= \frac{1}{h_{r}} f \bigl( \bigl\vert d(x,E_{k_{r}}) - d \bigl(x,\{0\} \bigr) \bigr\vert \bigr) \\ &= \frac{f(p_{r})}{h_{r}} \geq\frac{1}{h_{r}} h_{r} \to1\quad ( \text{as } r \to \infty), \end{aligned}$$

and so $(E_{k}) \notin[\mathit {WN}_{\theta}^{f}]$. This is a contradiction to the assumption that $\mathit{WS}_{\theta} = [\mathit {WN}_{\theta}^{f}]$. Hence, *f* is bounded. □

We now study a relationship between Wijsman strong Cesàro summability with respect to a modulus and Wijsman lacunary strong convergence with respect to a modulus.

### Theorem 2.11

*Let* $\theta= (k_{r})$ *be a lacunary sequence and* *f* *be a modulus*. *If* $1 <\liminf_{r} q_{r} \leq\limsup_{r} q_{r} < \infty$, *then* $[\mathit {WN}_{\theta }^{f}] = [\mathit {Ww}^{f}]$.

### Proof

Suppose that $\liminf_{r} q_{r} >1$, then there exists $\delta > 0$ such that $q_{r} \geq1+\delta$ for sufficiently large *r*. Let $(E_{k}) \in[\mathit {Ww}^{f}]$. For each $x \in M$, we have

2.1 $$ \begin{aligned}[b] &\frac{1}{h_{r}}\sum_{k \in I_{r}} f \bigl( \bigl\vert d(x,E_{k}) - d(x,E) \bigr\vert \bigr) \\ &\quad = \frac{1}{h_{r}}\sum_{k=1}^{k_{r}} f \bigl( \bigl\vert d(x,E_{k}) - d(x,E) \bigr\vert \bigr) - \frac{1}{h_{r}}\sum_{k=1}^{k_{r-1}} f \bigl( \bigl\vert d(x,E_{k}) - d(x,E) \bigr\vert \bigr) \\ &\quad =\frac{k_{r}}{h_{r}} \Biggl( \frac{1}{k_{r}}\sum _{k=1}^{k_{r}} f \bigl( \bigl\vert d(x,E_{k}) - d(x,E) \bigr\vert \bigr) \Biggr) - \frac{k_{r-1}}{h_{r}} \Biggl( \frac {1}{k_{r-1}}\sum_{k=1}^{k_{r-1}} f \bigl( \bigl\vert d(x,E_{k}) - d(x,E) \bigr\vert \bigr) \Biggr). \end{aligned} $$

Since $h_{r} = k_{r} - k_{r-1}$, we have

2.2 $$\begin{aligned} \frac{k_{r}}{h_{r}}\leq\frac{1+\delta}{\delta} \quad\text{and} \quad \frac{k_{r-1}}{h_{r}}\leq\frac{1}{\delta} \quad \text{for sufficiently large } r. \end{aligned}$$

Using (2.2) in (2.1), we have $\frac{1}{h_{r}}\sum_{k \in I_{r}} f(\vert d(x,E_{k}) - d(x,E)\vert ) \to0$ as the terms $\frac{1}{k_{r}}\sum_{k =1}^{k_{r}} f(\vert d(x, E_{k}) - d(x,E)\vert )$ and $\frac{1}{k_{r-1}}\sum_{k =1}^{k_{r-1}} f(\vert d(x,E_{k}) - d(x,E)\vert )$ both tends to 0 as $r \to\infty$. Hence, $(E_{k}) \in[\mathit {WN}_{\theta}^{f}]$.

Now suppose that $\limsup_{r}q_{r} < \infty$, then there exists $G>0$ such that $q_{r} < G$ for all $r \geq1$. Letting $(E_{k}) \in[\mathit {WN}_{\theta}^{f}]$, $x \in M$ and $\varepsilon> 0$ we can find $r_{0}$ such that for every $r \geq r_{0}$

$$N_{r} = \frac{1}{h_{r}}\sum_{ I_{r}} f \bigl( \bigl\vert d(x,E_{k}) - d(x,E) \bigr\vert \bigr) < \varepsilon. $$

We can also choose a number $L > 0$ such that $N_{r} \leq L $ for all *r*. Now let *n* be any integer with $k_{r-1} < n \leq k_{r}$, where $r > r_{0}$. Then, for each $x \in M $,

$$\begin{aligned} &\frac{1}{n}\sum_{k =1}^{n} f \bigl( \bigl\vert d(x,E_{k}) - d(x,E) \bigr\vert \bigr) \\ &\quad \leq\frac{1}{k_{r-1}}\sum_{k =1}^{k_{r}} f \bigl( \bigl\vert d(x,E_{k}) - d(x,E) \bigr\vert \bigr) \\ &\quad =\frac{1}{k_{r-1}} \biggl( \sum_{k \in I_{1}} f \bigl( \bigl\vert d(x,E_{k}) - d(x,E) \bigr\vert \bigr) + \cdots+\sum _{k \in I_{r}} f \bigl( \bigl\vert d(x,E_{k}) - d(x,E) \bigr\vert \bigr) \biggr) \\ &\quad =\frac{1}{k_{r-1}} \Biggl( \sum_{r=1}^{r_{0}} \sum_{k \in I_{r}} f \bigl( \bigl\vert d(x,E_{k}) - d(x,E) \bigr\vert \bigr) + \sum_{r=r_{0}+1}^{r} \sum_{k \in I_{r}} f \bigl( \bigl\vert d(x,E_{k}) - d(x,E) \bigr\vert \bigr) \Biggr) \\ &\quad < \frac{1}{k_{r-1}} \Biggl(\sum_{r=1}^{r_{0}} \sum_{k \in I_{r}} f \bigl( \bigl\vert d(x,E_{k}) - d(x,E) \bigr\vert \bigr) + \varepsilon(k_{r} - k_{r_{0}}) \Biggr) \\ &\quad = \frac{1}{k_{r-1}}(h_{1}N_{1}+ h_{2}N_{2}+ \cdots+h_{r_{0}}N_{r_{0}}) + \frac {1}{k_{r-1}} \varepsilon(k_{r} - k_{r_{0}}) \\ &\quad \leq\frac{1}{k_{r-1}} \Bigl(\sup_{1 \leq k \leq r_{0} }N_{k} \Bigr) k_{r_{0}} + \frac{1}{k_{r-1}}\varepsilon(k_{r} - k_{r_{0}}) \\ &\quad < L\frac{ k_{r_{0}}}{k_{r-1}} + \varepsilon G, \end{aligned}$$

which yields $(E_{k}) \in[\mathit {Ww}^{f}]$. □

## *f*-Wijsman lacunary statistical convergence

### Definition 3.1

Let $(M,\rho)$ be a metric space, *f* be an unbounded modulus and $\theta= (k_{r})$ be a lacunary sequence. A sequence $(E_{k})$ of non-empty closed subsets of *M* is said to be *f*-Wijsman lacunary statistically convergent to a non-empty closed subset *E* of *M*, or $\mathit{WS}_{\theta }^{f}$-convergent to *E*, if, for each $x \in M$ and for each $\varepsilon > 0$,

$$\lim_{r \to\infty}\frac{1}{f(h_{r})}f \bigl( \bigl\vert \bigl\{ k \in I_{r} : \bigl\vert d(x,E_{k}) - d(x,E) \bigr\vert \geq \varepsilon \bigr\} \bigr\vert \bigr) =0. $$

In this case, we write $\mathit{WS}_{\theta}^{f}-\lim E_{k} = E$ or $E_{k} \to E(\mathit{WS}_{\theta}^{f})$. The set of all sequences which are *f*-Wijsman lacunary statistically convergent is denoted by $\mathit{WS}_{\theta}^{f}$.

### Remark 3.2

The concept of *f*-Wijsman lacunary statistical convergence reduces to that of Wijsman lacunary statistical convergence when modulus is the identity mapping.

### Theorem 3.3

*Every Wijsman convergent sequence is* *f*-*Wijsman lacunary statistically convergent*, *however*, *the converse need not be true*.

### Proof

In view of the fact that finite sets have zero *f*-density, for any unbounded modulus *f*, it is easy to see that if a sequence is Wijsman convergent then it is *f*-Wijsman lacunary statistically convergent for any unbounded modulus *f*. For the converse part, let $M = \mathbb{R}$, $f(x) = x^{p}$, $0 < p \leq1$ and $(E_{k})$ be defined as

$$E_{k}= \textstyle\begin{cases} [ 2,h_{r} ] , &\text{if $k \geq2$ and $k \in I_{r}$ is a square,} \\ \{1\}, &\text{otherwise}, \end{cases} $$

where $\theta= (k_{r})$ is a lacunary sequence. This sequence is not Wijsman convergent, but, for each $x \in M$ and for each $\varepsilon> 0$,

$$\begin{aligned} \frac{1}{f(h_{r})}f \bigl( \bigl\vert \bigl\{ k \in I_{r} : \bigl\vert d(x, E_{k}) - d \bigl(x,\{1\} \bigr) \bigr\vert \geq \epsilon \bigr\} \bigr\vert \bigr)&\leq\frac{f(\sqrt{h_{r}})}{f(h_{r})} \\ &= \frac{(\sqrt{h_{r}})^{p}}{(h_{r})^{p}} = \frac{1}{(h_{r})^{p-p/2}} \to0 \quad (\text{as } r \to\infty) \end{aligned}$$

and hence, $(E_{k})$ is *f*-Wijsman lacunary statistically convergent to the set $E = \{1\}$. □

### Remark 3.4

In view of the above theorem it is clear that the notion of *f*-Wijsman lacunary statistical convergence is a generalization of the usual notion of the Wijsman convergence of sequences of sets.

### Theorem 3.5

*Every* *f*-*Wijsman lacunary statistically convergent sequence is Wijsman lacunary statistically convergent*.

### Proof

Suppose $(E_{k})$ is *f*-Wijsman lacunary statistically convergent to *E*. Let $x \in M$ and $\varepsilon> 0$. Then, for each positive integer *m*, there exists $r_{o} \in\mathbb{N}$ such that for $r \geq r_{o}$, we have

$$ f \bigl( \bigl\vert \bigl\{ k \in I_{r} : \bigl\vert d(x,E_{k}) - d(x,E) \bigr\vert \geq\epsilon \bigr\} \bigr\vert \bigr) \leq\frac{1}{m}f(h_{r}) \leq \frac{1}{m} mf \biggl( \frac{h_{r}}{m} \biggr) = f \biggl(\frac{h_{r}}{m} \biggr) $$

and since *f* is increasing, we have

$$\frac{1}{h_{r}} \bigl\vert \bigl\{ k \in I_{r} : \bigl\vert d(x,E_{k}) - d(x,E) \bigr\vert \geq \epsilon \bigr\} \bigr\vert \leq\frac{1}{m} . $$

Hence, $(E_{k})$ is Wijsman lacunary statistically convergent to *E*. □

### Remark 3.6

It seems that the converse of the above theorem need not hold, but right now we are not in a position to prove it. It is, therefore, left as an open problem.

We now establish a relationship between *f*-Wijsman lacunary statistical convergence and Wijsman lacunary strong convergence with respect to a modulus.

Maddox [31] showed the existence of an unbounded modulus *f* for which there is a positive constant *c* such that $f(xy) \geq cf(x)f(y)$, for all $x \geq0$, $y \geq0$. Using this we have the following.

### Theorem 3.7

*Let* $(M, \rho)$ *be a metric space and* $\theta= (k_{r})$ *be a lacunary sequence*, *then*

- *For any unbounded modulus* *f* *for which* $\lim_{t \to\infty}\frac{f(t)}{t} > 0$ *and there is a positive constant* *c* *such that* $f(xy)\geq cf(x)f(y)$ *for all* $x\geq0$, $y \geq0$,
  (i) $E_{k} \to E[\mathit {WN}_{\theta}^{f}]$ *implies* $E_{k} \to E(\mathit{WS}_{\theta}^{f})$,
  (ii) $[\mathit {WN}_{\theta}^{f}]$ *is a proper subset of* $\mathit{WS}_{\theta}^{f}$.
- $(E_{k} )\in L_{\infty}^{\prime}$ *and* $E_{k} \to E(\mathit{WS}_{\theta }^{f})$ *imply* $E_{k} \to E[\mathit {WN}_{\theta}^{f}]$, *for any unbounded modulus* *f*.
- $[\mathit {WN}_{\theta}^{f}] \cap L_{\infty}^{\prime} = \mathit{WS}_{\theta}^{f} \cap L_{\infty}^{\prime}$ *for any unbounded modulus* *f* *for which* $\lim_{t \to\infty}\frac{f(t)}{t} > 0$ *and there is a positive constant* *c* *such that* $f(xy)\geq cf(x)f(y)$ *for all* $x\geq0$, $y \geq0$.

### Proof

(a) (i) For any sequence $(E_{k})$, for each $x \in M$ and $\epsilon > 0$, by the definition of modulus function (ii) and (iii) we have

$$\begin{aligned} \frac{1}{h_{r}} \sum_{k \in I_{r}} f \bigl( \bigl\vert d(x,E_{k}) - d(x,E) \bigr\vert \bigr) &\geq \frac{1}{h_{r}} f \biggl(\sum_{k \in I_{r}} \bigl\vert d(x,E_{k}) - d(x,E) \bigr\vert \biggr) \\ & \geq\frac{1}{h_{r}} f \biggl(\mathop{\sum_{k \in I_{r}}}_{ \vert d(x,E_{k}) - d(x,E) \vert \geq\epsilon} \bigl\vert d(x,E_{k}) - d(x,E) \bigr\vert \biggr) \\ &\geq\frac{1}{h_{r}}f \bigl( \bigl\vert \bigl\{ k \in I_{r} : \bigl\vert d(x,E_{k}) - d(x,E) \bigr\vert \geq \varepsilon \bigr\} \bigr\vert \epsilon \bigr) \\ &\geq\frac{c}{h_{r}}f \bigl( \bigl\vert \bigl\{ k \in I_{r} : \bigl\vert d(x,E_{k}) - d(x,E) \bigr\vert \geq \varepsilon \bigr\} \bigr\vert \bigr) f ( \epsilon ) \\ &= \frac{c}{h_{r}}\frac{f (\vert \{ k \in I_{r} : d(x,E_{k}) - d(x,E) \geq \varepsilon\}\vert )}{f(h_{r})} f ( h_{r} )f ( \epsilon ), \end{aligned}$$

from which it follows that $(E_{k}) \in \mathit{WS}_{\theta}^{f}$ as $(E_{k}) \in [\mathit {WN}_{\theta}^{f}]$ and $\lim_{r \to\infty}\frac{f(h_{r})}{h_{r}} > 0$.

(ii) In order to show that the inclusion $[\mathit {WN}_{\theta}^{f}] \subset \mathit{WS}_{\theta}^{f}$ is proper, let $\theta= (k_{r})$ be a lacunary sequence and *f* be an unbounded modulus such that $\lim_{t \to\infty}\frac{f(t)}{t} > 0$ and there is a positive constant *c* such that $f(xy)\geq cf(x)f(y)$ for all $x\geq0$, $y\geq0$. Consider the sequence $(E_{k})$ such that $E_{k}$ to be $\{1\}, \{2\},\ldots,\{[\sqrt{h_{r}}]\} $ at the first $[\sqrt{h_{r}}]$ integers in $I_{r}$, and $E_{k} = \{0\}$ otherwise. Note that $(E_{k})$ is not bounded. Also, for each $x \in M$ and $\epsilon> 0$,

$$\begin{aligned}& \begin{aligned} \frac{1}{f(h_{r})}f \bigl( \bigl\vert \bigl\{ k \in I_{r} : \bigl\vert d(x,E_{k}) - d \bigl(x,\{0\} \bigr) \bigr\vert \geq \varepsilon \bigr\} \bigr\vert \bigr) &= \frac{f([\sqrt{h_{r}} ])}{f(h_{r})} \\ &=\frac{f([\sqrt{h_{r}} ])}{[\sqrt{h_{r}} ]}\times\frac{h_{r}}{f(h_{r})}\times \frac{[\sqrt{h_{r}} ]}{h_{r}} \\ &\to0 \quad \text{as } r \to\infty, \quad \text{because} \end{aligned} \\ & \lim_{r \to\infty}\frac{f([\sqrt{h_{r}} ])}{[\sqrt{h_{r}} ]}, \lim_{r \to\infty} \frac{f(h_{r})}{h_{r}} \text{ are positive\quad and}\quad \lim_{r \to \infty} \frac{[\sqrt{h_{r}} ]}{h_{r}} = 0. \end{aligned}$$

Thus, $E_{k} \to\{0\}(\mathit{WS}_{\theta}^{f})$. On the other hand, for $x=0$,

$$\begin{aligned} &\frac{1}{h_{r}}\sum_{k \in I_{r}} f \bigl( \bigl\vert d(x,E_{k}) - d \bigl(x,\{0\} \bigr) \bigr\vert \bigr) \\ &\quad = \frac{1}{h_{r}}\mathop{\sum_{k \in I_{r}}}_{k_{r-1} < k \leq k_{r-1}+([\sqrt{h_{r}}])}f \bigl( \bigl\vert d(x,E_{k}) - d \bigl(x,\{0\} \bigr) \bigr\vert \bigr) \\ &\quad = \frac{1}{h_{r}} \bigl[f \bigl( \bigl\vert \vert x-1\vert -\vert x-0\vert \bigr\vert \bigr) + f \bigl( \bigl\vert \vert x-2\vert - \vert x-0\vert \bigr\vert \bigr)+\cdots+ f \bigl( \bigl\vert \bigl\vert x-[\sqrt{h_{r}}] \bigr\vert -\vert x-0\vert \bigr\vert \bigr) \bigr] \\ &\quad = \frac{1}{h_{r}} \bigl[ f(1) + f(2) + \cdots+ f \bigl([ \sqrt{h_{r}} ] \bigr) \bigr] \\ &\quad \geq\frac{f(1+2+\cdots+[\sqrt{h_{r}} ])}{h_{r}} \\ &\quad = \frac{f (\frac{[\sqrt{h_{r}} ]([\sqrt{h_{r}} ]+1)}{2} )}{h_{r}} \\ &\quad \geq c\frac{f([\sqrt{h_{r}} ])f (\frac{[\sqrt{h_{r}} ]+1}{2} )}{h_{r}} \\ &\quad =c \biggl(\frac{f([\sqrt{h_{r}} ])}{[\sqrt{h_{r}} ]} \biggr) \biggl( \frac {f (\frac{[\sqrt{h_{r}} ]+1}{2} )}{\frac{[\sqrt{h_{r}} ]+1}{2}} \biggr) \biggl( \frac{[\sqrt{h_{r}} ] (\frac{[\sqrt{h_{r}} ]+1}{2} )}{h_{r}} \biggr) \\ &\quad >0 \quad \text{as } c, \lim_{r \to \infty}\frac{f([\sqrt{h_{r}} ]}{[\sqrt{h_{r}} ]}, \lim _{r \to \infty}\frac{f (\frac{[\sqrt{h_{r}} ]+1}{2} )}{\frac{[\sqrt {h_{r}} ]+1}{2}} , \text{and } \lim_{r \to\infty } \frac{[\sqrt{h_{r}} ] (\frac{[\sqrt{h_{r}} ]+1}{2} )}{h_{r}} \end{aligned}$$

are positive. Therefore, $E_{k} \nrightarrow\{0\}[\mathit {WN}_{\theta}^{f}]$.

(b) Suppose that $E_{k} \to E(\mathit{WS}_{\theta}^{f})$ and $(E_{k}) \in L_{\infty}^{\prime}$, say $\vert d(x,E_{k}) - d(x,E) \vert \leq G$, for each $x \in M$ and for all $k \in\mathbb{N}$, where $G = \sup_{k}\vert d(x,E_{k})\vert + d(x,E)$. Given $\epsilon> 0$ and for each $x \in M$, we have

$$\begin{aligned} &\frac{1}{h_{r}} \sum_{k \in I_{r}} f \bigl( \bigl\vert d(x,E_{k}) - d(x,E) \bigr\vert \bigr) \\ &\quad = \frac{1}{h_{r}} \mathop{\sum_{k \in I_{r}}}_{ \vert d(x,E_{k}) - d(x,E)\vert \geq\epsilon} f \bigl( \bigl\vert d(x,E_{k}) - d(x,E) \bigr\vert \bigr) \\ &\quad\quad{} +\frac{1}{h_{r}} \mathop{\sum_{k \in I_{r}}}_{\vert d(x,E_{k}) - d(x,E)\vert < \epsilon} f \bigl( \bigl\vert d(x,E_{k}) - d(x,E) \bigr\vert \bigr) \\ &\quad \leq \frac{1}{h_{r}} \bigl\vert \bigl\{ k \in I_{r} : \bigl\vert d(x,E_{k}) - d(x,E) \bigr\vert \geq \varepsilon \bigr\} \bigr\vert f(G) + \frac{1}{h_{r}}h_{r}f(\epsilon). \end{aligned}$$

Taking the limit on both sides as $r \to\infty$, we get

$$\lim_{r \to\infty}\frac{1}{h_{r}} \sum _{k \in I_{r}} f \bigl( \bigl\vert d(x,E_{k}) - d(x,E) \bigr\vert \bigr) = 0, $$

in view of Theorem 3.5 and the fact that *f* is increasing.

(c) This is an immediate consequence of (a) and (b). □

### Remark 3.8

The example given in part (a) of the above theorem shows that the boundedness condition cannot be omitted from the hypothesis of part (b).

### Remark 3.9

If we take $f(x) = x$ in Theorem 3.7, we obtain Theorem 1 of Ulusu and Nuray [22].

Quite recently, Bhardwaj *et al.* have established the following lemmas [unpublished].

### Lemma 3.10

*Let* $(M,\rho)$ *be a metric space*; *f*, *g* *be unbounded moduli*, $(E_{k})$ *be a sequence of non*-*empty closed sets in* *M* *and* $E, F \subset M$ *be non*-*empty closed*. *We have*

(i) *The* *f*-*Wijsman statistical limit is unique whenever it exists*.
(ii) *Moreover*, *two different methods of Wijsman statistical convergence are always compatible*, *which means that if* $\mathit{WS}^{f}-\lim E_{k} = E$ *and* $\mathit{WS}^{g}-\lim E_{k} = F$ *then* $E = F$.

### Lemma 3.11

*For any modulus* *f*, *we have* $[\mathit {Ww}] \subset[\mathit {Ww}^{f}]$.

### Lemma 3.12

*Let* *f* *be a modulus such that* $\lim_{t \to\infty}\frac{f(t)}{t} > 0$, *then* $[\mathit {Ww}^{f}] \subset[\mathit {Ww}]$.

### Lemma 3.13

*Let* $(M,\rho)$ *be a metric space*, *f* *be an unbounded modulus such that* $\lim_{t \to\infty}\frac{f(t)}{t}>0$ *and there is a positive constant*

$$c \textit{ such that}\quad f( xy)\geq cf(x)f(y)\quad \textit{for all }x\geq0, y\geq 0. $$

*Then for any non*-*empty closed subsets* $E, E_{k} \subset M$:

(i) $(E_{k})$ *is* *f*-*Wijsman statistically convergent to* *E* *if it is Wijsman strongly Cesàro summable to* *E*, *with respect to* *f*.
(ii) *If* $(E_{k})$ *is bounded and* *f*-*Wijsman statistically convergent to* *E* *then it is Wijsman strongly Cesàro summable to* *E* *with respect to f*.

We now study the inclusions $\mathit{WS}_{\theta}^{f} \subset \mathit{WS}^{f}$ and $\mathit{WS}^{f} \subset \mathit{WS}_{\theta}^{f}$ under certain restrictions on *θ* and *f*.

### Lemma 3.14

*For any lacunary sequence* *θ*, *and unbounded modulus* *f* *for which* $\lim_{t \to\infty}\frac{f(t)}{t} > 0$ *and there is a positive constant* *c* *such that* $f(xy)\geq cf(x)f(y)$ *for all* $x\geq0$, $y \geq0$, *we have* $\mathit{WS}^{f} \subset \mathit{WS}_{\theta}^{f}$ *if and only if* $\liminf_{r} q_{r} >1$.

### Proof

(Sufficiency). If $\liminf_{r} q_{r} >1$, then there exists $\beta > 0$ such that $q_{r} \geq1+\beta$ for sufficiently large *r*. Since $h_{r} = k_{r} - k_{r-1}$, we have

$$\frac{h_{r}}{k_{r}} \geq\frac{\beta}{1+\beta} $$

for sufficiently large *r*. If $E_{k} \to E(\mathit{WS}^{f})$, then, for each $\epsilon> 0$, for each $x \in M$ and sufficiently large *r*, we have

$$\begin{aligned} &\frac{1}{f(k_{r})}f \bigl( \bigl\vert \bigl\{ k \leq k_{r} : \bigl\vert d(x,E_{k}) - d(x,E) \bigr\vert \geq \varepsilon \bigr\} \bigr\vert \bigr) \\ &\quad \geq\frac{f (\vert \{ k \in I_{r} : \vert d(x,E_{k}) - d(x,E)\vert \geq \varepsilon\}\vert )}{f(k_{r})} \\ &\quad = \frac{f(h_{r})}{f(k_{r})} \biggl( \frac{f (\vert \{ k \in I_{r} : \vert d(x,E_{k}) - d(x,E)\vert \geq \varepsilon\}\vert )}{f(h_{r})} \biggr) \\ &\quad = \biggl(\frac{f(h_{r})}{h_{r}} \biggr) \biggl(\frac{k_{r}}{f(k_{r})} \biggr) \biggl( \frac{h_{r}}{k_{r}} \biggr) \biggl( \frac{f (\vert \{ k \in I_{r} : \vert d(x,E_{k}) - d(x,E)\vert \geq \varepsilon\}\vert )}{f(h_{r})} \biggr) \\ &\quad \geq \biggl(\frac{f(h_{r})}{h_{r}} \biggr) \biggl(\frac{k_{r}}{f(k_{r})} \biggr) \biggl(\frac{\beta}{1+\beta} \biggr) \biggl( \frac{f (\vert \{ k \in I_{r} : \vert d(x,E_{k}) - d(x,E)\vert \geq \varepsilon\}\vert )}{f(h_{r})} \biggr) . \end{aligned}$$

This proves the sufficiency.

(Necessity). Assume that $\liminf_{r} q_{r} = 1$. Proceeding as in Lemma 2.1 of [39], we can select a subsequence $(k_{r(j)})$ of *θ* satisfying

$$ \frac{k_{r(j)}}{k_{r(j) -1}} < 1+ \frac{1}{j} \quad \text{and} \quad \frac{k_{r(j)-1}}{k_{r(j-1)}} > j , \quad \text{where } r(j)\geq r(j-1)+2. $$

Define a sequence $(E_{k})$ as follows:

$$E_{k} = \textstyle\begin{cases} \{ (x,y) \in\mathbb{R\times R} : x^{2} + (y-1)^{2} = \frac{1}{k^{4}}\}, &\mbox{if }k \in I_{r(j)}, \text{for some } j= 1,2,3,\ldots,\\ \{(0,0)\}, & \mbox{otherwise.} \end{cases} $$

It is easy to see that the sequence $(E_{k})$ is bounded because for each $(x,y) \in\mathbb{R\times R} $, the sequence $(d((x,y), E_{k}))$ given as below is bounded.

$$d \bigl((x,y),E_{k} \bigr) = \textstyle\begin{cases} \vert \frac{x^{2} +(y-1)^{2} - \frac{1}{k^{4}}}{\sqrt{x^{2} +(y-1)^{2}} + \frac{1}{k^{2}} }\vert , &\mbox{if }k \in I_{r(j)}, \text{for some } j= 1,2,3,\ldots,\\ \sqrt{x^{2}+y^{2}}, & \mbox{otherwise.} \end{cases} $$

Also, it is shown in Lemma 1 of [22] that $(E_{k}) \notin [\mathit {WN}_{\theta}]$ but $(E_{k}) \in [\mathit {Ww}]$. Thus, in view of Theorems 2.7 and 3.7, we have $(E_{k}) \notin \mathit{WS}_{\theta}^{f}$. On the other hand, it follows from Lemmas 3.11 and 3.13 that $(E_{k}) \in \mathit{WS}^{f}$. Hence, $\mathit{WS}^{f} \not\subset \mathit{WS}_{\theta}^{f}$. But this is a contradiction to the assumption that $\mathit{WS}^{f} \subset \mathit{WS}_{\theta}^{f}$. This contradiction shows that our assumption is wrong. Hence, $\liminf_{r} q_{r} > 1$. □

### Lemma 3.15

*For any lacunary sequence* *θ*, *and unbounded modulus* *f* *for which* $\lim_{t \to\infty}\frac{f(t)}{t} > 0$ *and there is a positive constant* *c* *such that* $f(xy)\geq cf(x)f(y)$ *for all* $x\geq0$, $y \geq0$, *we have* $\mathit{WS}_{\theta}^{f} \subset \mathit{WS}^{f}$ *if and only if* $\limsup_{r} q_{r} < \infty$.

### Proof

(Sufficiency). If $\limsup_{r} q_{r} < \infty$, then there is a $K > 0$ such that $q_{r} < K$ for all *r*. Now, suppose that $E_{k} \to E(\mathit{WS}_{\theta}^{f})$ and $\lim_{r \to\infty}\frac{f(h_{r})}{h_{r}} = l'$. Therefore, for given $\epsilon> 0$ and for each $x \in M$, there exists $r_{o} \in \mathbb{N}$ such that for all $r > r_{o}$

$$\begin{aligned} &\frac{f(h_{r})}{h_{r}} < l' + \epsilon, \\ &\text{and} \quad\frac{1}{f(h_{r})}f \bigl( \bigl\vert \bigl\{ k \in I_{r} : \bigl\vert d(x,E_{k}) - d(x,E) \bigr\vert \geq \varepsilon \bigr\} \bigr\vert \bigr) < \epsilon. \end{aligned}$$

Let $N_{r} =\vert \{ k \in I_{r} :\vert d(x,E_{k}) - d(x,E)\vert \geq\epsilon\}\vert $. Using this notation, we have

$$\frac{f(N_{r})}{f(h_{r})} < \epsilon \quad \text{for all } r > r_{o}. $$

Now, let $G = \max\{ f(N_{1}), f(N_{2}), \ldots, f(N_{r_{o}})\}$ and let *n* be an integer such that $k_{r-1} < n \leq k_{r}$, where $r > r_{o}$ then we can write

$$\begin{aligned} &\frac{1}{f(n)}f \bigl( \bigl\vert \bigl\{ k \leq n : \bigl\vert d(x,E_{k}) - d(x,E) \bigr\vert \geq \varepsilon \bigr\} \bigr\vert \bigr) \\ &\quad \leq\frac{1}{f(k_{r-1})}f \bigl( \bigl\vert \bigl\{ k \leq k_{r} : \bigl\vert d(x,E_{k}) - d(x,E) \bigr\vert \geq \varepsilon \bigr\} \bigr\vert \bigr) \\ &\quad =\frac{1}{f(k_{r-1})}f( N_{1} + N_{2} + \cdots+ N_{r_{o}} + N_{r_{o}+1} + \cdots+ N_{r}) \\ &\quad \leq\frac{1}{f(k_{r-1})} \bigl(f(N_{1}) + f(N_{2})+ \cdots+ f(N_{r_{o}})+ f(N_{r_{o}+1})+ \cdots+ f(N_{r}) \bigr) \\ &\quad \leq\frac{r_{o}G}{f(k_{r-1})} + \frac{1}{f(k_{r-1})} \bigl[f(N_{r_{o}+1}) + \cdots+ f(N_{r}) \bigr] \\ &\quad =\frac{r_{o}G}{f(k_{r-1})} + \frac{1}{f(k_{r-1})} \biggl[\frac{f(h_{r_{o}+1})}{h_{r_{o}+1}} \frac {f(N_{r_{o}+1})}{f(h_{r_{o}+1})}h_{r_{o}+1}+ \cdots+ \frac{f(h_{r})}{h_{r}} \frac{f(N_{r})}{f(h_{r})}h_{r} \biggr] \\ &\quad < \frac{r_{o}G}{f(k_{r-1})} + \frac{1}{f(k_{r-1})} \bigl[ \bigl(l'+\epsilon \bigr)\epsilon h_{r_{o}+1}+\cdots+ \bigl(l'+\epsilon \bigr) \epsilon h_{r} \bigr] \\ &\quad =\frac{r_{o}G}{f(k_{r-1})} + \frac{1}{f(k_{r-1})} \epsilon \bigl(l'+ \epsilon \bigr) [h_{r_{o}+1} + \cdots + h_{r} ] \\ &\quad =\frac{r_{o}G}{f(k_{r-1})} + \frac{1}{f(k_{r-1})} \epsilon \bigl(l'+ \epsilon \bigr) [k_{r} - k_{r_{o}} ] \\ &\quad < \frac{r_{o}G}{f(k_{r-1})} + \epsilon \bigl(l'+\epsilon \bigr) \biggl[ \frac{k_{r}}{f(k_{r-1})} \biggr] \\ & \quad = \frac{r_{o}G}{f(k_{r-1})} + \epsilon \bigl(l'+\epsilon \bigr) \frac{1}{\frac{f(k_{r-1})}{k_{r-1}}}\frac {k_{r}}{k_{r-1}} \\ &\quad =\frac{r_{o}G}{f(k_{r-1})} + \epsilon \bigl(l'+\epsilon \bigr)q_{r}\frac{1}{\frac{f(k_{r-1})}{k_{r-1}}} \\ &\quad < \frac{r_{o}G}{f(k_{r-1})} + \epsilon \bigl(l'+\epsilon \bigr)K \frac{1}{\frac{f(k_{r-1})}{k_{r-1}}} , \end{aligned}$$

from which the sufficiency follows immediately, in view of the fact that $\lim_{r \to \infty} \frac{f(k_{r-1})}{k_{r-1}} > 0$.

(Necessity). Suppose that $\limsup_{r} q_{r} = \infty$. Following Lemma 2.2 of [39], we can select a subsequence $(k_{r(j)})$ of lacunary sequence *θ* such that $q_{r(j)} > j$. Define a bounded sequence $(E_{k})$ by

$$E_{k} = \textstyle\begin{cases} \{1\}, &\mbox{if }k_{r(j)-1} < k \leq2k_{r(j)-1}, \text{for some } j= 1,2,3,\ldots,\\ \{0\}, & \mbox{otherwise.} \end{cases} $$

It is shown in Lemma 2 of [22] that $(E_{k}) \in[\mathit {WN}_{\theta}]$ but $(E_{k}) \notin[\mathit {Ww}]$. By Theorems 2.4 and 3.7, we conclude that $(E_{k}) \in \mathit{WS}_{\theta}^{f}$, but $(E_{k}) \notin \mathit{WS}^{f}$, in view of Lemmas 3.12 and 3.13. Hence, $\mathit{WS}_{\theta}^{f} \not\subset \mathit{WS}^{f}$. But this is a contradiction to the assumption that $\mathit{WS}_{\theta}^{f} \subset \mathit{WS}^{f}$. This contradiction shows that $\limsup_{r} q_{r} < \infty$. □

Combining Lemmas 3.14 and 3.15 we have the following.

### Theorem 3.16

*For any lacunary sequence* *θ*, *and unbounded modulus* *f* *for which* $\lim_{t \to\infty}\frac{f(t)}{t} > 0$ *and there is a positive constant* *c* *such that* $f(xy)\geq cf(x)f(y)$ *for all* $x\geq0$, $y \geq0$, *we have* $\mathit{WS}_{\theta}^{f} = \mathit{WS}^{f}$ *if and only if* $1 <\liminf_{r} q_{r} \leq\limsup_{r} q_{r} < \infty$.

## Uniqueness of $\mathit{WS}_{\theta}^{f}$-limit

It is easy to see that, for any fixed *θ*, the $\mathit{WS}_{\theta }$-limit is unique. It is possible, however, for a sequence, even a bounded one, to have different $\mathit{WS}_{\theta}$-limits for different *θ*’s. This can be seen by applying Theorem 1 of Ulusu and Nuray [22] to the sequence $(E_{k})$ defined as follows:

$$E_{k} = \textstyle\begin{cases} \{1\}, &\text{if $p(k)$ is even},\\ \{0\}, & \text{if $p(k)$ is odd}, \end{cases} $$

where $p(k) = n$, if $n! < k \leq(n+1)!$ and $\theta_{1} = ((2r)!)$, $\theta_{2} = ((2r+1)!)$.

It is observed that, for each $x \in M$,

$$\frac{1}{h_{r+1}}\sum_{I_{r+1}} \bigl\vert d(x,E_{k}) - d \bigl(x,\{0\} \bigr) \bigr\vert = \frac{(2r+1)! - (2r)!}{(2r+2)! - (2r)!} \to0\quad (\mbox{as } r \to\infty), $$

from which it follows that $(E_{k}) \in[\mathit {WN}_{\theta_{1}}]$ with $[\mathit {WN}_{\theta _{1}}]-\lim E_{k} = \{0\}$, hence, $\mathit{WS}_{\theta_{1}}-\lim E_{k} = \{0\}$ and also

$$\frac{1}{h_{r}}\sum_{I_{r}} \bigl\vert d(x,E_{k}) - d \bigl(x,\{1\} \bigr) \bigr\vert = \frac{(2r)! - (2r-1)!}{(2r+1)! - (2r-1)!} \to0 \quad (\mbox{as } r \to \infty), $$

from which it follows that $(E_{k}) \in[\mathit {WN}_{\theta_{2}}]$ with $[\mathit {WN}_{\theta _{2}}]-\lim E_{k} = \{1\}$ and hence, $\mathit{WS}_{\theta_{2}}-\lim E_{k} = \{1\}$.

In case of $\mathit{WS}_{\theta}$-convergence, Ulusu and Nuray [22] showed that this situation cannot if the sequence is Wijsman statistically convergent. We now establish a similar result in the case of $\mathit{WS}_{\theta}^{f}$-convergence. First we observe that it is also possible for a sequence to have different $\mathit{WS}_{\theta}^{f}$-limits for different *θ*’s. To illustrate this, let us take $f(x) = 2x$, $\theta_{1} = ((2r)!)$, $\theta_{2} = ((2r+1)!)$ and consider the sequence $(E_{k})$ just defined above for which $[\mathit {WN}_{\theta_{1}}]-\lim E_{k} = \{0\}$ and $[\mathit {WN}_{\theta _{2}}]-\lim E_{k} = \{1\}$. Now by applying Theorem 2.4 and Theorem 3.7, we see that $\mathit{WS}_{\theta_{1}}^{f}-\lim E_{k} = \{0\}$ and $\mathit{WS}_{\theta_{2}}^{f}-\lim E_{k} = \{1\}$.

In the next theorem we investigate certain conditions under which this situation cannot occur.

### Theorem 4.1

*For any two lacunary sequences* $\theta_{1}$ *and* $\theta_{2}$, *if* $(E_{k}) \in \mathit{WS}^{f} \cap(\mathit{WS}_{\theta_{1}}^{f} \cap \mathit{WS}_{\theta_{2}}^{f})$, *then* $\mathit{WS}_{\theta_{1}}^{f}-\lim E_{k} = \mathit{WS}_{\theta_{2}}^{f}-\lim E_{k}$, *where* *f* *is an unbounded modulus function such that*

$$\bigl\vert f(x) - f(y) \bigr\vert = f \bigl(\vert x-y \vert \bigr), \quad \textit{for all } x\geq0, y \geq0. $$

To prove this theorem we need the following lemma.

### Lemma 4.2

*For any lacunary sequence* *θ*, *if* $(E_{k}) \in \mathit{WS}^{f} \cap \mathit{WS}_{\theta}^{f}$, *then* $\mathit{WS}_{\theta}^{f}-\lim E_{k} = \mathit{WS}^{f}-\lim E_{k}$, *where* *f* *is an unbounded modulus function such that*

$$\bigl\vert f(x) - f(y) \bigr\vert = f \bigl(\vert x-y \vert \bigr), \quad \textit{for all } x\geq0, y \geq0. $$

### Proof

Suppose $\mathit{WS}^{f}-\lim E_{k} = E$, $\mathit{WS}_{\theta}^{f}-\lim E_{k} =F$ and $E \neq F$. Since $E \neq F$, therefore there exists at least one $x \in M$ such $x \in E$ but $x \notin F$ or $x \in F$ but $x \notin E$. Without loss of generality we may suppose that $x \in E$ but $x \notin F$. Now, clearly *x* cannot be a limit point of *F*, because if *x* becomes a limit point of *F* then $x \in F$ as *F* is closed. Now, let $\varepsilon> 0$ be such that $0 < \varepsilon< \frac{\vert d(x,E) - d(x,F)\vert }{2}$. Using the definition of modulus (iii) and (ii), we have

$$\begin{aligned} &\frac{f (\vert \{ k \leq n : \vert d(x,E) - d(x,F)\vert \geq 2\varepsilon\}\vert )}{f(n)} \\ &\quad \leq\frac{f (\vert \{ k \leq n : \vert d(x,E_{k}) - d(x,E)\vert \geq \varepsilon\}\vert )}{f(n)} \\ &\quad\quad{} +\frac{f (\vert \{ k \leq n : \vert d(x,E_{k}) - d(x,F)\vert \geq \varepsilon\}\vert )}{f(n)}. \end{aligned}$$

Taking the limit as $n \to\infty$ on both sides, we get

4.1 $$ \begin{aligned} &1 \leq0 + \lim_{n \to\infty}\frac{f (\vert \{ k \leq n : \vert d(x,E_{k}) - d(x,F)\vert \geq \varepsilon\}\vert )}{f(n)} \leq1, \\ &\text{and hence},\quad \lim_{n \to\infty}\frac{f (\vert \{ k \leq n : \vert d(x,E_{k}) - d(x,F)\vert \geq \varepsilon\}\vert )}{f(n)} = 1. \end{aligned} $$

Now consider the $k_{m}$th term of the sequence

4.2 $$\begin{aligned} &\bigl( \bigl(f(n) \bigr)^{-1} f \bigl( \bigl\vert \bigl\{ k \leq n : \bigl\vert d(x,E_{k}) - d(x,F) \bigr\vert \geq \varepsilon \bigr\} \bigr\vert \bigr) \bigr). \\ &\frac{1}{f(k_{m})}f \bigl( \bigl\vert \bigl\{ k \leq k_{m} : \bigl\vert d(x,E_{k}) - d(x,F) \bigr\vert \geq \varepsilon \bigr\} \bigr\vert \bigr) \\ &\quad = \frac{1}{f(k_{m})}f \Biggl( \Biggl\vert \Biggl\{ k \in\bigcup _{r=1}^{m} I_{r} : \bigl\vert d(x,E_{k}) - d(x,F) \bigr\vert \geq \varepsilon \Biggr\} \Biggr\vert \Biggr) \\ &\quad = \frac{1}{f(k_{m})}f \Biggl(\sum_{r=1}^{m} \bigl\vert \bigl\{ k \in I_{r} : \bigl\vert d(x,E_{k}) - d(x,F) \bigr\vert \geq \varepsilon \bigr\} \bigr\vert \Biggr) \\ &\quad \leq\frac{1}{f(k_{m})}\sum_{r=1}^{m}f \bigl( \bigl\vert \bigl\{ k \in I_{r} : \bigl\vert d(x,E_{k}) - d(x,F) \bigr\vert \geq \varepsilon \bigr\} \bigr\vert \bigr) \\ &\quad =\frac{1}{f(k_{m})}\sum_{r=1}^{m}f(h_{r}) \frac{1}{f(h_{r})}f \bigl( \bigl\vert \bigl\{ k \in I_{r} : \bigl\vert d(x,E_{k}) - d(x,F) \bigr\vert \geq \varepsilon \bigr\} \bigr\vert \bigr). \end{aligned}$$

Also, in view of the choice of unbounded modulus *f*, we have

4.3 $$\begin{aligned} \sum_{r=1}^{m} f(h_{r}) &= f(h_{1})+f(h_{2})+ \cdots+f(h_{m}) \\ &=f(k_{1} - k_{o})+f(k_{2} - k_{1})+ \cdots+f(k_{m}-k_{m-1}) \\ &=f \bigl(\vert k_{1} - k_{o}\vert \bigr)+f \bigl( \vert k_{2} - k_{1}\vert \bigr)+\cdots+f \bigl(\vert k_{m}-k_{m-1}\vert \bigr) \\ &= \bigl\vert f(k_{1}) - f(k_{o}) \bigr\vert + \bigl\vert f(k_{2}) - f(k_{1}) \bigr\vert +\cdots+ \bigl\vert f(k_{m}) - f(k_{m-1}) \bigr\vert \\ &=f(k_{1}) - f(k_{o})+f(k_{2}) - f(k_{1})+\cdots+f(k_{m}) - f(k_{m-1}) \\ &=f(k_{m}). \end{aligned}$$

Now, using (4.3) in (4.2), we have

4.4 $$ \frac{1}{f(k_{m})}f \bigl( \bigl\vert \bigl\{ k \leq k_{m} : \bigl\vert d(x,E_{k}) - d(x,F) \bigr\vert \geq \varepsilon \bigr\} \bigr\vert \bigr) \leq\frac{1}{\sum_{r=1}^{m} f(h_{r}) }\sum _{r=1}^{m}f(h_{r})t_{r}, $$

where $t_{r} = (f(h_{r}))^{-1}f (\vert \{ k \in I_{r} : \vert d(x,E_{k}) - d(x,F)\vert \geq \varepsilon\}\vert ) \to0$ because $E_{k} \to F(\mathit{WS}_{\theta}^{f})$. Since *θ* is a lacunary sequence and *f* being modulus is increasing, the term on the right hand side of (4.4) is a regular weighted mean transformation of $t=(t_{r})$, and therefore, it, too, tends to zero as $r \to\infty$. Thus,

$$\frac{1}{f(k_{m})}f \bigl( \bigl\vert \bigl\{ k \leq k_{m} : \bigl\vert d(x,E_{k}) - d(x,F) \bigr\vert \geq \varepsilon \bigr\} \bigr\vert \bigr) \to0 \quad \text{as } m \to \infty. $$

Also, since $( (f(k_{m}))^{-1}f (\vert \{ k \leq k_{m} : \vert d(x,E_{k}) - d(x,F)\vert \geq \varepsilon\}\vert ) ) $ is a subsequence of sequence $( (f(n))^{-1}f (\vert \{ k \leq n : \vert d(x,E_{k}) - d(x,F)\vert \geq \varepsilon\}\vert ) ) $, we conclude that

$$\bigl(f(n) \bigr)^{-1}f \bigl( \bigl\vert \bigl\{ k \leq n : \bigl\vert d(x,E_{k}) - d(x,F) \bigr\vert \geq \varepsilon \bigr\} \bigr\vert \bigr) \nrightarrow1. $$

But this is a contradiction to (4.1). This contradiction shows that $E = F$. □

### Proof of Theorem 4.1

By Lemma 4.2, we have

4.5 $$\begin{aligned} &\mathit{WS}^{f}-\lim E_{k} = \mathit{WS}_{\theta_{1}}^{f}-\lim E_{k} \quad\text{and} \end{aligned}$$

4.6 $$\begin{aligned} &\mathit{WS}^{f}-\lim E_{k} = \mathit{WS}_{\theta_{2}}^{f}-\lim E_{k}. \end{aligned}$$

Therefore, from (4.5) and (4.6) we have

$$\mathit{WS}_{\theta_{1}}^{f}-\lim E_{k} = \mathit{WS}_{\theta_{2}}^{f}- \lim E_{k} . $$

 □

If we take $f(x) =x$ in Theorem 4.1 we obtain the following result which contains Theorem 3 of Ulusu and Nuray [22].

### Corollary 4.3

*For any two lacunary sequences* $\theta_{1}$ *and* $\theta_{2}$, *if* $(E_{k}) \in \mathit{WS} \cap( \mathit{WS}_{\theta_{1}} \cap \mathit{WS}_{\theta_{2}})$, *then* $\mathit{WS}_{\theta_{1}}-\lim E_{k} = \mathit{WS}_{\theta_{2}}-\lim E_{k}$.

In the next theorem we show that if a sequence $(E_{k})$ is $\mathit{WS}_{\theta }^{f}$-convergent and $\mathit{WS}_{\theta}^{g}$-convergent then $\mathit{WS}_{\theta}^{f}-\lim E_{k} = \mathit{WS}_{\theta}^{g}-\lim E_{k}$, under certain conditions on the unbounded moduli *f* and *g*.

### Theorem 4.4

*For any lacunary sequence* *θ*, *if* $(E_{k}) \in(\mathit{WS}^{f} \cap \mathit{WS}_{\theta}^{f})\cap(\mathit{WS}^{g} \cap \mathit{WS}_{\theta}^{g})$, *then* $\mathit{WS}_{\theta}^{f}-\lim E_{k} = \mathit{WS}_{\theta}^{g}-\lim E_{k}$, *where* *f* *and* *g* *are unbounded moduli such that*

$$\begin{aligned} &\bigl\vert f(x) - f(y) \bigr\vert = f \bigl(\vert x-y \vert \bigr) \quad \textit{and} \\ &\bigl\vert g(x) - g(y) \bigr\vert = g \bigl(\vert x-y \vert \bigr), \quad \textit{for all } x\geq0, y \geq0. \end{aligned}$$

### Proof

By Lemma 4.2, we have

4.7 $$ \begin{aligned} &\mathit{WS}^{f}-\lim E_{k} = \mathit{WS}_{\theta}^{f}-\lim E_{k} \quad\text{and} \\ &\mathit{WS}^{g}-\lim E_{k} = \mathit{WS}_{\theta}^{g}-\lim E_{k}. \end{aligned} $$

But according to Lemma 3.10, we have

4.8 $$ \mathit{WS}^{f}-\lim E_{k} = \mathit{WS}^{g}-\lim E_{k}. $$

Therefore, from (4.7) and (4.8), we have

$$\mathit{WS}_{\theta}^{f}-\lim E_{k} = \mathit{WS}_{\theta}^{g}- \lim E_{k}. $$

 □
